# The Organogermanium Compound 3-(Trihydroxygermyl) Propanoic Acid (THGP) Suppresses Inflammasome Activation Via Complexation with ATP

**DOI:** 10.3390/ijms232113364

**Published:** 2022-11-01

**Authors:** Junya Azumi, Yasuhiro Shimada, Tomoya Takeda, Hisashi Aso, Takashi Nakamura

**Affiliations:** 1 Asai Germanium Research Institute Co., Ltd. Suzuranoka 3-131, Hakodate 042-0958, Hokkaido, Japan; 2 Laboratory of Animal Health Science, Graduate School of Agricultural Science, Tohoku University, Sendai 980-8572, Miyagi, Japan

**Keywords:** inflammation, inflammasome, THGP, Ge-132, organogermanium

## Abstract

Inflammasome activity is a key indicator of inflammation. The inflammasome is activated by pathogen-associated molecular patterns (PAMPs) and damage-associated molecular patterns (DAMPs), which activate the p38-NF-κB pathway and promote IL-1β transcription (signaling step 1). Next, extracellular adenosine triphosphate (ATP) activates the inflammasome (a protein complex consisting of a signal recognition protein, an adapter protein, and Caspase-1) and secretion of inflammatory cytokines such as IL-1β (signaling step 2). Inflammasome activation causes excessive inflammation, leading to inflammasome-active diseases such as atherosclerosis and type 2 diabetes. A hydrolysate of the organogermanium compound Ge-132, 3-(Trihydroxygermyl) propanoic acid (THGP) can form a complex with a cis-diol structure. We investigated the inhibitory effect of THGP on inflammasome activity in human THP-1 monocytes. THGP inhibited IL-1β secretion and caspase-1 activation (signaling step 2) in an ATP-dependent manner. On the other hand, THGP did not suppress IL-1β secretion induced by only lipopolysaccharide (LPS) stimulation. In addition, as IL-6 is an ATP-independent inflammatory cytokine, THGP did not decrease its secretion. THGP also suppressed pyroptosis, which is a caspase-1 activity-dependent form of cell death. Therefore, THGP is expected to become a new therapeutic or prophylactic agent for inflammasome-associated diseases.

## 1. Introduction

The inflammasome is a protein complex that is a key indicator of inflammation. The inflammasome is composed of recognition proteins such as NLRP3, NLRC4, NLRP1, and AIM2; an adapter protein called ASC; and the execution protein Caspase-1 [1,2]. NLRP3, which is a major inflammasome component, is expressed in cells such as macrophages, keratinocytes, and osteoblasts [3]. Activation of the inflammasome leads to the release of the inflammatory cytokines IL-1β and IL-18 [4,5]. The mechanism of inflammasome activation can be broadly divided into the following two signaling steps. Pathogen-associated molecular patterns (PAMPs) are exogenous substances such as viruses and endotoxins, and damage-associated molecular patterns (DAMPs) are endogenous substances such as uric acid and cholesterol crystals [6]. They activate the p38-NF-κB pathway [6], and the transcription factor NF-κB promotes the transcription of inflammatory cytokines such as IL-1β and IL-18 and inflammasome constituent proteins such as NLRP3 and Caspase-1. Subsequently, in the second signaling step, intracellular adenosine triphosphate (ATP) is released in response to inflammatory stimulation, and extracellular ATP is secreted from neighboring cells. ATP then signals via P2X and P2Y receptors by an autocrine or paracrine mechanism [7]. At this time, Ca^2+^ influx and ROS generation are stimulated by ATP [8]. In addition, when the inflammasome is activated by ATP, caspase-1 is also activated [9]. Activated caspase-1 cleaves pro-IL-1β transcribed in response to signaling step 1, and IL-1β is released extracellularly. Activation of the inflammasome plays an important role as a defense mechanism; however, excessive activation leads to various diseases, such as Alzheimer’s disease and arteriosclerosis [10].

It has been reported that the organogermanium compound Ge-132 and its hydrolysate 3-(trihydroxygermyl)propanoic acid (THGP) have various physiological activities [11,12,13]. THGP has also been reported to form complexes with cis-diol structures [14,15,16]. Furthermore, we have reported that THGP suppresses ATP-induced calcium influx in normal human epithelial keratinocytes by forming a complex with ATP [17].

Therefore, we hypothesize that THGP suppresses inflammasome activation by forming a complex with ATP. In this study, we investigate the suppressive effect of THGP on lipopolysaccharide (LPS)- and ATP-induced inflammasome activity using macrophage-differentiated human THP-1 monocytes.

## 2. Results

### 2.1. IL-1β Secretion from THP-1 Cells Was Suppressed by THGP

**Figure 1:**
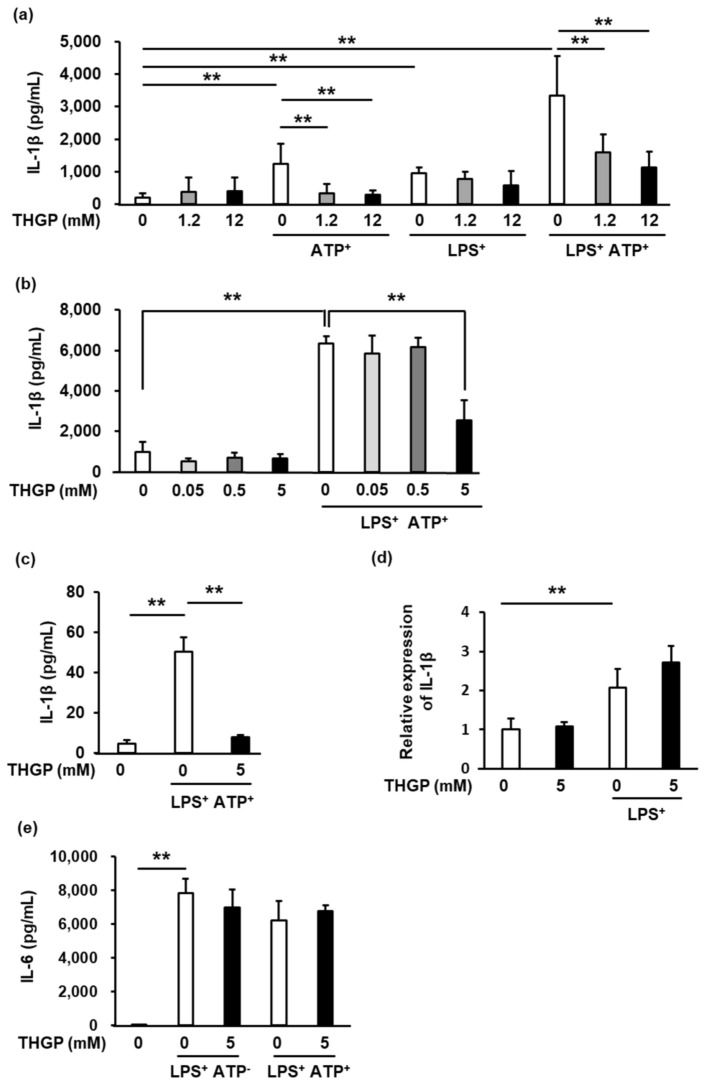
The suppressive effect of THGP on the inflammasome via signaling step 2. (**a**) The secretion of IL-1β from THP-1 cells was measured by ELISA after stimulation with 10 μg/mL LPS, 5 mM ATP, or the combination of the two. THGP was added at a concentration of 0–12 mM. (**b**) The secretion of IL-1β from THP-1 cells was measured by ELISA after stimulation with 10 μg/mL LPS and 1 mM ATP. THGP was added at a concentration of 0–5 mM. (**c**) The secretion of IL-1β from RAW 264.7 cells was measured by ELISA after stimulation with 10 μg/mL LPS and 1 mM ATP. THGP was added at a concentration of 5 mM. (**d**) The effect of THGP on the mRNA expression of IL-1β in THP-1 cells treated with 10 μg/mL LPS and 5 mM THGP was investigated by real-time RT–PCR. (**e**) The secretion of IL-6 from THP-1 cells was measured by ELISA after stimulation with 10 μg/μL LPS and/or 5 mM ATP. THGP was added at a concentration of 5 mM. The mean and S.D. are shown. n = 6. ** *p* < 0.01.

We investigated the inhibitory effect of THGP on inflammasome activity in human THP-1 monocytes that were induced to differentiate into macrophage-like cells by phorbol 12-myristate 13-acetate (PMA). IL-1β secretion was induced by ATP alone, LPS alone, or ATP and LPS together (ATP/LPS) and assessed by ELISA (Figure 1a). THGP at 1.2 mM and 12 mM had an inhibitory effect on IL-1β secretion upon stimulation with ATP alone and with the combination of ATP and LPS. However, upon stimulation with LPS alone, THGP did not suppressed IL-1β secretion. These results suggest that THGP suppresses the secretion of IL-1β induced by ATP. Furthermore, we investigated the optimal concentration of THGP (Figure 1b). At 5 mM, but not at 0.05 or 0.5 mM, THGP significantly inhibited IL-1β secretion by approximately 30%. Therefore, we hypothesized that the optimal concentration of THGP is between 0.5 mM and 1.2 mM. In addition, 5 mM THGP suppressed the secretion of IL-1β from RAW 264.7 murine macrophage cells (Figure 1c). In contrast, the mRNA expression of IL-1β, which is involved in signaling step 1, was not affected by THGP after stimulation with LPS alone (Figure 1d). Furthermore, as IL-6 is an ATP-independent inflammatory cytokine, IL-6 was secreted in response to LPS stimulation regardless of whether ATP was added. THGP did not inhibit IL-6 secretion upon stimulation with either LPS alone or LPS/ATP (Figure 1e). These results suggested that THGP suppressed IL-1β secretion in an ATP-dependent manner.

### 2.2. Caspase-1 Activity Was Suppressed by THGP

**Figure 2:**
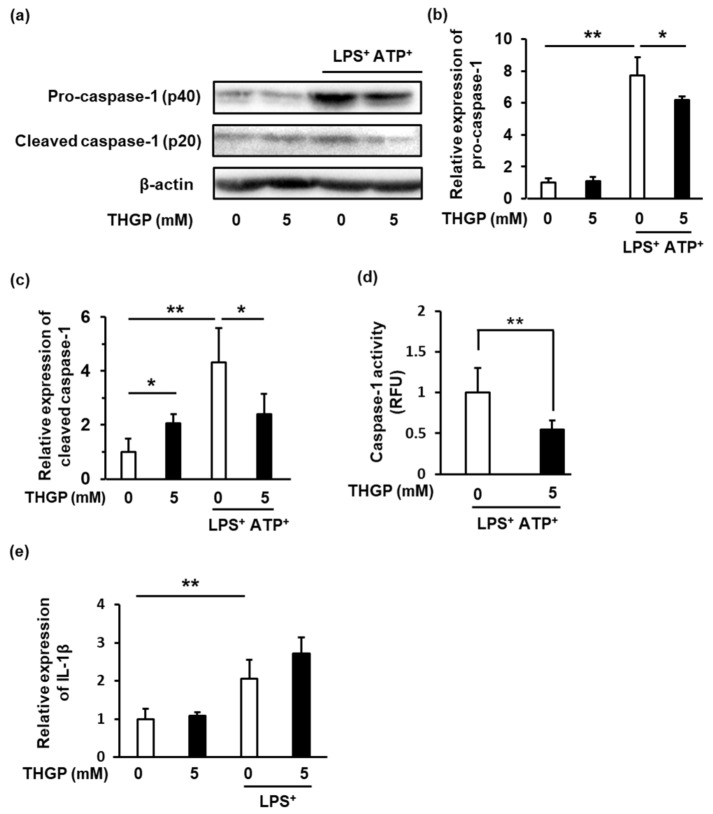
The effect of THGP on caspase-1 expression in THP-1 cells. (**a**–**c**) The effect of THGP on the expression of Caspase-1 in THP-1 cells treated with or without 10 μg/mL LPS and 1 mM ATP was investigated by western blotting. β-Actin was used as an internal standard to quantify the expres-sion of each protein. (**d**) Caspase-1 activity in THP-1 cells treated with 10 μg/mL LPS, 1 mM ATP and/or 5 mM THGP was measured using a Caspase-Glo® 1 Inflammasome Assay Kit. (**e**) The effect of THGP on the mRNA expression of IL-1β in THP-1 cells treated with 10 μg/ml LPS and 5 mM THGP was investigated by real-time RT–PCR. The mean and S.D. are shown. n = 6. * *p* < 0.01, ** *p* < 0.05.

Caspase-1 activation is required for inflammasome activation and the secretion of IL-1β. Therefore, we investigated the effect of THGP on the expression of caspase-1 in THP-1 cells after LPS/ATP stimulation using western blotting (Figure 2a–c). THGP significantly suppressed the expression of pro-caspase-1 by 20% and the expression of cleaved caspase-1 by 56% in LPS/ATP-stimulated THP-1 cells. However, THGP increased the expression of cleaved caspase-1 2-fold in THP-1 cells not stimulated with LPS/ATP. In addition, caspase-1 activity was suppressed by THGP in LPS/ATP-stimulated THP-1 cells.

### 2.3. THGP Suppressed Inflammasome Activity by Forming a Complex with ATP

**Figure 3:**
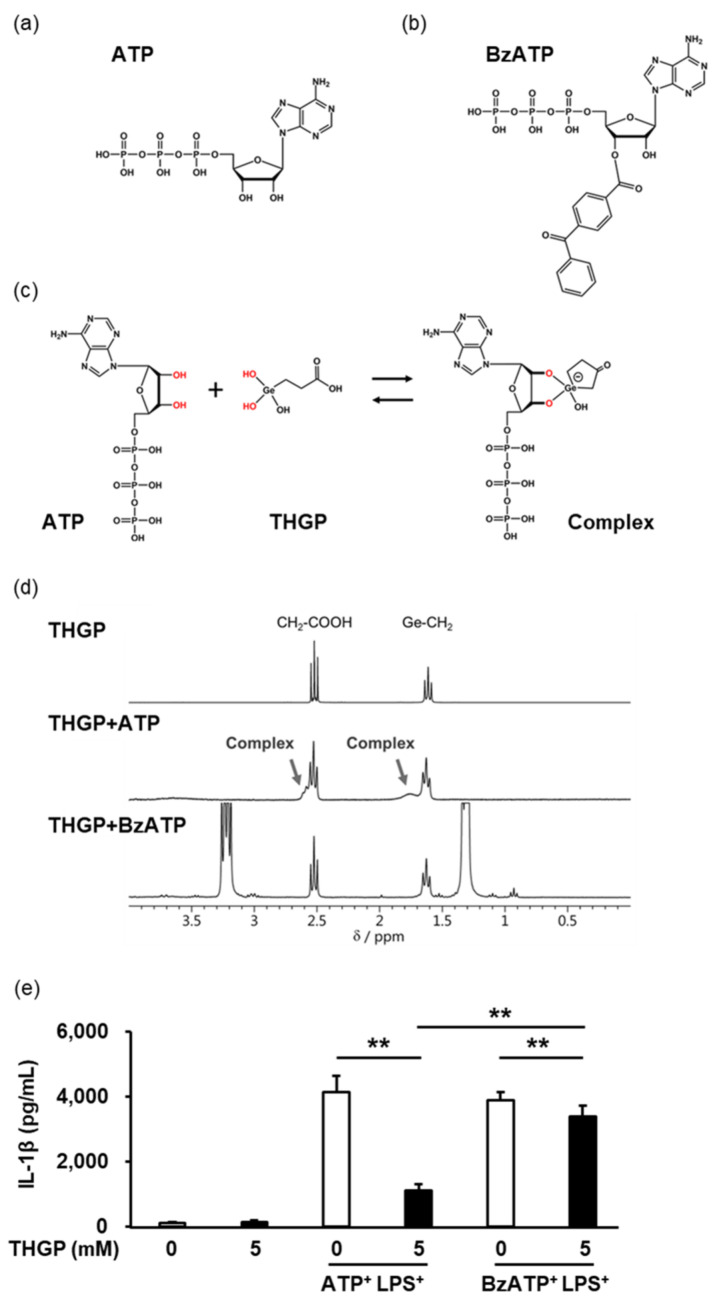
The inhibitory effect of THGP on inflammasome activity is dependent on ATP. The structural formulas and structures of ATP (**a**) and BzATP (**b**) and the estimated structure of the complex formed by ATP and THGP (**c**). (**d**) Complex formation between THGP and ATP or BzATP was investigated by ^1^H-NMR. (**e**) After stimulation with a combination of LPS with ATP or BzATP, the content of IL-1β secreted from THP-1 cells was measured by ELISA. The results and S.D. are shown. The mean and S.D. are shown. n = 6. ** *p* < 0.01.

It was previously reported that THGP can interact with ATP to form a complex with a cis-diol structure (Figure 3c) [17]. Therefore, a comparative study was conducted using ATP and 2′(3′)-O-(4-benzoylbenzoyl)adenosine 5′-triphosphate triethylammonium (BzATP). BzATP is an agonist of P2 × 7R, a main receptor for ATP, and has an approximately 10 times higher affinity for P2X7R than ATP. The structure of BzATP is very similar to that of ATP except that BzATP has a benzoyl group on one side of its diol structure (Figure 3a, b). To evaluate complex formation between THGP and ATP or between THGP and BzATP, ^1^H-NMR analysis was performed (Figure 3d). When THGP and ATP were mixed at a 1:1 ratio, new signals derived from a complex formed between the two compounds were observed near the original signal in addition to each intrinsic spectrum (arrows in Figure 3d). In contrast, when THGP and BzATP were mixed, a new signal was not observed. Therefore, BzATP could not form a complex with THGP. These results show that THGP can specifically interact with ATP to form a complex with a cis-diol structure. Subsequently, the secretion of IL-1β from THP-1 cells stimulated with a combination of LPS and ATP or BzATP was examined (Figure 3e). The suppressive effect of THGP on IL-1β secretion was stronger upon stimulation with ATP (suppression rate of 75%) than upon stimulation with BzATP (suppression rate of 14%). These results suggested that THGP suppressed inflammasome activity in an ATP-dependent manner. However, THGP had a weak suppressive effect on IL-1β secretion induced by BzATP. Therefore, THGP may also suppress inflammasome activity by an ATP-independent manner.

### 2.4. THGP Suppressed The Induction of Pyroptosis by LPS/ATP Stimulation

**Figure 4:**
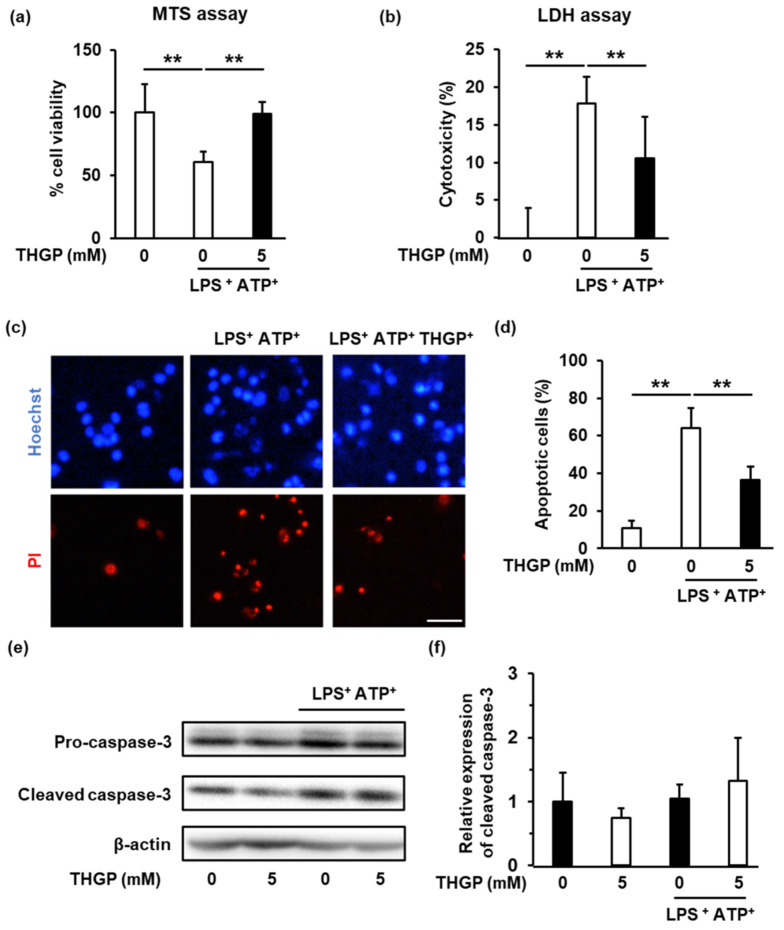
Suppressive effect of THGP on pyroptosis induced by LPS/ATP. (**a**) The viability of THP-1 cells stimulated with 10 μg/mL LPS and 1 mM ATP was investigated using the MTS assay. (**b**) Cytotoxicity following LPS/ATP stimulation was investigated using the LDH assay. (**c,d**) The number of dead cells was determined by Hoechst/PI staining. Red and blue cells are dead (PI) and live cells (Hoechst), respectively (Scale bar: 100 μm). (**e,f**) The expression of caspase-3 was measured by western blotting. β-Actin was used as an internal standard to quantify the expression of each protein. The mean and S.D. are shown. n = 6. ** *p* < 0.01.

Pyroptosis is a caspase-1-dependent form of cell death that is induced by inflammasome activation. On the other hand, apoptosis is a form of caspase-3-dependent cell death induced by extracellular stimulation [18]. Since THGP suppressed caspase-1 activity induced by ATP/LPS stimulation, we examined the effect of THGP on pyroptosis. First, an MTS assay was performed to assess cell viability. The cell viability decreased to 60% upon stimulation with LPS and ATP, and an improvement in cell viability was observed upon the addition of THGP (Figure 4a). Subsequently, cytotoxicity was assessed by the LDH assay, and the results revealed that THGP increased cell viability by approximately 40% (Figure 4b). Finally, propidium iodide (PI)/Hoechst staining was performed to assess cell death. The cell death rate following LPS/ATP stimulation was approximately 60%, whereas the percentage of dead cells decreased to 36% upon the addition of THGP (Figure 4c,d). Subsequently, to confirm that THGP acted in a caspase-1-dependent manner, the activity of the apoptosis marker caspase-3 was investigated (Figure 4e). Caspase-3 activity was not changed by LPS/ATP stimulation, and the addition of THGP did not affect caspase-3 activity. The above results show that THGP suppresses caspase-1-dependent pyroptosis induced by LPS/ATP stimulation.

## 3. Discussion

The inflammasome is associated with a wide variety of diseases, such as metabolic pathologies (obesity, type 2 diabetes, atherosclerosis), cardiovascular diseases (ischemic and non-ischemic heart disease), inflammatory conditions (liver diseases, inflammatory bowel diseases, gut microbiome disorders, rheumatoid arthritis), and neurological disorders (Parkinson’s disease, Alzheimer’s disease, multiple sclerosis, amyotrophic lateral sclerosis, etc.) [19]. The NLRP3-ASC-Caspase-1 complex plays a major role in inflammasome activation [20]. Therefore, the suppressing activity of the inflammasome complex is important for maintaining good health.

Asaigermanium^®^ (Ge-132) is a crystalline polymer that is hydrolyzed to THGP when dissolved in water [21]. Previous in vitro and in vivo studies by our laboratory and collaborative research center have revealed the various functions of Ge-132 and THGP [12,22,23,24,25]. In addition, the function of complexes formed by THGP with cis-diol structures has been clarified in vitro. For example, through its cis-diol structure, the complex formed by THGP and ATP inhibits the binding of ATP to P2YRs and then suppresses Ca^2+^ influx in normal human keratinocytes [17]. As L-DOPA, a precursor of melanin, has a cis-diol structure, THGP forms a complex with it and suppresses the production of melanin in B16 4A5 melanoma cells [26]. In this study, we discovered for the first time that THGP suppresses the activity of the inflammasome by forming a complex with ATP. ATP is called a “danger signal”. High levels of extracellular ATP are passively released from necrotic cells, and ATP acts as a pro-inflammatory factor, activating the NLRP3 inflammasome through binding to the ionotropic P2X7 receptor [22]. We revealed that THGP suppressed step 2 of inflammasome activation by forming a complex with ATP (Figure 1, Figure 2 and Figure 3). This step plays an important role in the secretion of IL-1β. Therefore, it is important to identify therapeutic strategies and drugs that can suppress this process to prevent inflammasome-related diseases. Various drugs that can inhibit step 2 of inflammasome activation, such as MCC950, CY-09, and KN62, have been identified [23]. Moreover, almost all inflammasome inhibitors act as antagonists of ATP receptors, e.g., P2X, P2Y, etc., or inhibit NLRP3 activity [24]. In contrast, THGP inhibits inflammasome activity through a unique mechanism. Whereas many inhibitors target inflammasome-related proteins, THGP targets ATP, a small molecule nucleotide and a principal component of the inflammasome. Since THGP has a unique mechanism of action, it is expected to have synergistic effects with inflammasome inhibitors via other mechanisms. In addition, THGP acts on its substrates and does not directly affect protein activity; therefore, the risk of side effects is very low [14].

In this study, it was determined that THGP suppresses ATP-mediated inflammasome activation and subsequent IL-1β secretion. As the secretion of the inflammatory cytokine IL-6 was not affected by ATP, THGP did not have an inhibitory effect on its secretion (Figure 1e). In contrast, THGP slightly inhibited IL-1β secretion and inflammasome activity induced by BzATP, an agonist of ATP; however, THGP could not form a complex with BzATP (Figure 3). These data indicate that THGP may suppress the inflammasome independent of signaling step 2. Indeed, in previous in vitro experiments, THGP was found to suppress the phosphorylation of NF-κB, p38, ERK, and JNK in mouse mammary gland cells to block inflammation induced by LPS. In addition, THGP has been reported to suppress the expression of TNF-α, IL-β, and IL-6 in the absence of ATP [25]. Thus, THGP may suppress the secretion of inflammatory cytokines in an ATP-independent manner. Further research on signaling step 1 is needed.

Severe acute respiratory syndrome coronavirus-2 (SARS-CoV-2) led to the COVID-19 pandemic in 2019, and COVID-19 is now widespread worldwide [27]. ORF3a, which is a structural protein of SARS-CoV-2, has been reported to activate the inflammasome [28,29]. In addition to inducing the secretion of IL-1β, ORF3a promotes the secretion of inflammatory cytokines such as IL-6 and TNF-α [30,31]. Excessive cytokine secretion induces a cytokine storm and aggravates inflammation. Therefore, THGP may effectively inhibit the cytokine storm during SARS-CoV-2 infection by suppressing inflammasome activity. Several authors have noted that Ge-132 may prevent aggravation of COVID-19 induced by anti-oxidative stress and immune activation [32,33]. It has been also reported that Ge-132 suppresses influenza virus growth and reduces mouse mortality [34]. Most recently, it was revealed that THGP has dual effects in suppressing the growth of influenza virus A and the development of pneumonia by directly binding to the 5′-triphosphate RNA of influenza virus A [35]. SARS-CoV-2, unlike influenza virus, was not found to interact with THGP. However, it is still expected that THGP can interact with ORF3a of SARS-CoV-2 and ATP to exert an anti-inflammatory effect via suppression of inflammasome activation. Moreover, previous in vitro studies and clinical trials have shown that Ge-132 can treat inflammasome-related diseases, such as pneumonia, rheumatism, and supraclavicular arteriosclerosis [36,37]. Findings indicate that these effects may be due to natural killer (NK) cell activation and antioxidant effects and may also involve complex formation between ATP and THGP. While this study only demonstrated the mechanism underlying the inhibitory effect of THGP on inflammasome activity, it did not examine the effect of THGP on inflammasome-related disease. Further studies on these topics are needed.

Previous reports have clarified that THGP induces IFN-γ production and activates NK cells or macrophages [38,39]. However, the opposite effect was observed in our study. Our findings also showed that THGP alone increased the expression of cleaved-caspase-1. As pro-inflammatory macrophages (M1 macrophage) express higher levels of cleaved-caspase-1 [40], treatment with THGP alone may induce the polarization of macrophages toward the M1 phenotype. Ge-132 may also regulate immune balance. THGP binds its ligand in aqueous solution. It has been proven that the affinity of THGP for its ligand changes depending on the concentration of THGP and ligand [17]. In our study, we confirmed that the combination of 5 mM THGP and 5 mM ATP inhibited IL-1β secretion to a greater extent than the combination of 5 mM THGP and 1 mM ATP (70.1% vs. 26.7%) (data not shown). In short, THGP has a stronger effect in the presence of excessive amounts of ATP resulting from inflammation but may not exert its effect under normal conditions due to the low level of ATP. For these reasons, THGP may be a regulator of immune balance.

Ge-132, a polymer of THGP, has been widely studied worldwide for its bioactivity [41]. In addition, Ge-132 is used as a raw material for cosmetics and health foods worldwide [42]. The safety of the Ge-132 (commonly known as Asaigermanium^®^) used in this study, which was manufactured at the Asai Germanium Research Institute, was verified through various studies conducted in accordance with “Good Laboratory Practice Standards for Safety Studies on Drugs”, including tests of mutagenicity (unpublished data). The safety of Ge-132 in mice, rats, and dogs has been confirmed by multiple studies. For example, it was found that the NOAEL of Ge-132 is 270 mg/kg/day and that a single oral dose of 8500 mg/kg Ge-132 does not cause death in rats. In another study, daily administration of 5000 mg/kg Ge-132 did not cause any of the rats to die, and the maximum safe dose was estimated to be 1667 mg/kg [43,44]. Furthermore, it has been reported that oral Ge-132 is noncarcinogenic in mice and rats up to a concentration of 1,500 mg/kg/day [45,46]. A phase I study in Japan showed that in humans, Ge-132 is nontoxic up to a concentration of 75 mg/kg [47]. Furthermore, 1500 mg/day of Ge-132 was found to have no side effects in females in a clinical trial [36]. These results suggest that Ge-132 is very safe. Thus, it would be easier to gain approval for the use of Ge-132 in the clinic. Therefore, we expect that an increasing number of researchers will begin exploring the medical applications of Asaigermanium. However, while this study demonstrated the mechanism underlying the inhibitory effect of THGP on inflammasome activity, it did not examine its effect on inflammasome-related diseases. In the future, it will be necessary to examine the effects of THGP on inflammasome-related diseases.

**Figure 5:**
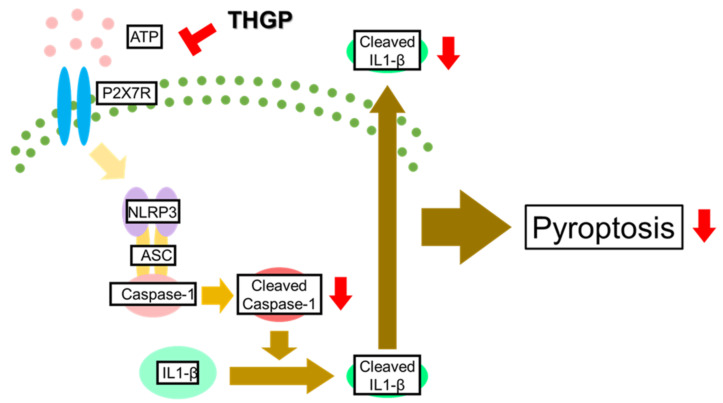
Mechanism by which THGP inhibits inflammasome activation induced by LPS/ATP. THGP suppresses the activity of Caspase-1 and the secretion of IL-1β by complexing with ATP in the presence of LPS/ATP. THGP also suppresses LPS/ATP-induced pyroptosis.

In conclusion, in this study, it was clarified that the complex formed between THGP and ATP inhibits inflammasome activity (Figure 5). Therefore, THGP is expected to be a therapeutic/prophylactic drug for various inflammasome-related diseases.

## 4. Materials and Methods

### 4.1. Preparation of THGP

Ge-132 was manufactured as a food ingredient, Asaigermanium^®^, by Asai Germanium Research Institute Co., Ltd. (Kawasaki, Japan) Ge-132 was dissolved in ultrapure water at a concentration of 500 mM, and the pH was adjusted to 7.05 with NaOH (Wako Pure Chemical Industries Ltd., Osaka, Japan). Then, THGP was sterilized by membrane filtration and stored at 4 °C.

### 4.2. Cell Culture

Human THP-1 monocytes and RAW 264.7 murine macrophages cells were purchased from Riken Cell Bank. The THP-1 cells were cultured in RPMI supplemented with 10% FBS (Nissui Pharmaceutical Co., Ltd., Tokyo, Japan) at 37 °C in 5% CO_2_. The THP-1 cells were passaged every 2–4 days at a ratio of 1:5–10. In the experiment, THP-1 cells were differentiated into macrophage-like cells by adding 1 μM PMA (Sigma–Aldrich Corp., St. Louis, MO, USA) to the cells at the time of seeding. The RAW 264.7 cells were cultured in DMEM supplemented with 10% FBS (Nissui Pharmaceutical Co., Ltd.) at 37 °C in 5% CO_2_. The RAW 264.7 cells were passaged every 2–4 days at a ratio of 1:5–10.

### 4.3. ELISA

THP-1 cells were seeded in a 24-well plate at a density of 1 × 10^5^ cells/well and cultured for 24 h in the presence of 1 μM PMA. Then, THGP was added at various concentrations, and the cells were cultured for 24 h. Furthermore, 10 μg/mL LPS (Sigma–Aldrich Corp., St. Louis, MO, USA) and THGP were added, and the cells were cultured for 24 h. Subsequently, the cells were treated with 1 or 5 mM ATP (Tokyo Chemical Industry Co., Ltd., Tokyo, Japan) or 100 μM BzATP (Wako Pure Chemical Industries Ltd.) for 2 h, and the supernatant was collected. The levels of cytokines were measured using a human IL-1β or IL-6 ELISA kit (Diaclone SAS, Besançon, France) according to the manufacturer’s protocol. All reagent concentrations noted are the final concentrations. The same applies to the subsequent experimental methods. RAW 264.7 cells were seeded in a 24-well plate at a density of 1 × 10^5^ cells/well. After 24 h, 5 mM THGP was added, and the cells were cultured for 24 h. Furthermore, 10 μg/mL LPS and THGP were added, and the cells were cultured for 24 h. Subsequently, the cells were treated with 1 mM ATP for 2 h. The supernatant was collected, and IL-1β levels were measured using a mouse IL-1β ELISA kit (Proteintech, Rosemont, IL, USA).

### 4.4. Western Blotting

THP-1 cells were seeded in 6 cm dishes at a density of 2 × 10^6^ cells/well and cultured for 24 h in the presence of 1 μM PMA. Then, 5 mM THGP was added, and the cells were cultured for 24 h. Furthermore, 10 μg/mL LPS and 5 mM THGP were added, and the cells were cultured for 24 h. In addition, 24 h after LPS was added, 1 mM ATP was added for 2 h. Then, protein was extracted using RIPA buffer. The protein concentration was quantified by the Bradford method (Bio–Rad Laboratories Inc., Hercules, CA, USA). Ten micrograms of protein were electrophoresed on a polyacrylamide gel. Primary antibodies against Caspase-1 (Santa Cruz), Caspase-3 (Cell Signaling), and β-Actin (Abcam Ltd., Cambridge, UK) were used. The primary antibodies were diluted to the recommended concentration. The secondary antibodies used corresponded to the primary antibodies (Abcam). Blocking buffers used were 5% skim milk (Morinaga Milk Industry Co., Ltd., Tokyo, Japan) in TBS-T. The antibodies were diluted with blocking buffer. β-Actin was used as an internal standard to quantify the expression of each protein.

### 4.5. Caspase-1 Activity

THP-1 cells were seeded in a 96-well plate at a concentration of 2 × 10^4^ cells/well and cultured for 24 h in the presence of 1 μM PMA. Then, 5 mM THGP was added, and the cells were cultured for 24 h. Furthermore, 10 μg/mL LPS and THGP were added, and the cells were cultured for 24 h. Subsequently, ATP (1 mM) was added for 24 h. The Caspase-Glo^®^ 1 Inflammasome Assay (Promega Corp., Madison, WI, USA) was used according to the manufacturer’s instructions.

### 4.6. H-NMR

The signals of THGP, ATP, or BzATP and their mixtures were measured by ^1^H-NMR using a Mercury Plus 300 MHz instrument (Agilent Technologies, Santa Clara, CA, USA). A 5 mm micro bottom tube (SP-501; Shigemi Co., Ltd., Tokyo, Japan) was used as the NMR tube. For measurement, the concentration of each sample was adjusted to a concentration of 50 mM and pH of 7.01, and the samples were mixed at a molar ratio of 1:1. Additionally, 25 mM (pH 7.0) phosphate buffer prepared with heavy water (Kanto Chemical Co., Inc., Tokyo, Japan) was used as the solvent. The light water (HOD set to 4.80 ppm) remaining in the heavy water solvent was used as an internal standard for chemical shifts. The measurement temperature was 25 °C, and the total number of integrations was 256.

### 4.7. Real Time RT–PCR

THP-1 cells were seeded in a 6-well plate at a concentration of 1 × 10^6^ cells/well and cultured for 24 h in the presence of 1 μM PMA. Then, 5 mM THGP was added, and the cells were cultured for 24 h. Furthermore, 10 μg/mL LPS and THGP were added, the cells were cultured for 24 h, and RNA was extracted using Isogen (Nippon Gene Co., Ltd., Tokyo, Japan) following the manufacturer’s protocol. The extracted RNA (1 μg) was reverse transcribed at 50 °C for 1 h and 95 °C for 5 min using Superscript III (Invitrogen, Life Technologies Corp., Carlsbad, CA, USA) and oligo(dT)20 primer. PCR was performed using TB Green Premix Ex Taq II (Tli RNaseH Plus) (Takara Bio Inc., Otsu, Japan) at 95 °C for 5 s and 60 °C for 30 s for 40 cycles. mRNA expression was normalized using RPS18 as an internal control. The primers are shown in Table 1.

### 4.8. Cell Viability Assay (MTS Assay)

THP-1 cells were seeded in a 96-well plate at a concentration of 1 × 10^4^ cells/well and cultured for 24 h in the presence of 1 μM PMA. Then, 5 mM THGP was added, and the cells were cultured for 24 h. Furthermore, 10 μg/mL LPS and THGP were added, and the cells were cultured for 24 h. Subsequently, ATP (1 mM) was added for 24 h. The experiments were performed a CellTiter 96^®^ AQueous One Solution Cell Proliferation Assay kit (Promega) according to the manufacturer’s protocol using.

### 4.9. LDH Assay

THP-1 cells were seeded in a 96-well plate at a concentration of 1 × 10^4^ cells/well and cultured for 24 h in the presence of 1 μM PMA. Then, 5 mM THGP was added, and the cells were cultured for 24 h. Furthermore, 10 μg/mL LPS and THGP were added, and the cells were cultured for 24 h. Subsequently, ATP (1 mM) was added for 24 h. The experiments were conducted using an LDH assay kit (Dojindo Laboratories) according to the manufacturer’s protocol.

### 4.10. PI-Hoechst Staining

A total of 1 × 10^6^ cells/well were seeded in a 6-well plate and cultured for 24 h in the presence of 1 μM PMA. Then, 5 mM THGP was added, and the cells were cultured for 24 h. Furthermore, 10 μg/mL LPS and THGP were added. Subsequently, ATP (1 mM) was added for 2 h. Then, the cells were stained by Propidium Iodide (Dojindo Laboratories) and Hoechst 33452 (Dojindo Laboratories). Hoechst 33452-stained cells were counted as total cells and Propidium Iodide-stained cells were counted as apoptotic cells.

### 4.11. Statistical Analysis

Statistical analyses were performed with Statcel-the Useful Add-in Forms in Excel, 4th ed (OMS Publishing Co., Ltd., Tokyo, Japan). The data were analyzed using two-tailed Student’s t-tests for two groups. Data were compared among more than two groups using one-way ANOVA and Dunnett’s test. *p* < 0.05 was considered to indicate a statistically significant difference.

## Figures and Tables

**Table 1:** Primers used for real-time RT–PCR.

	Forward	Reverse
*Il-1β*	TTACAGTGGCAATGAGGATGAC	GTCGGAGATTCGTAGCTGGAT
*Rps18*	TCAGCCTCTTCTCCTTCCTG	GGCTACAGGCTTGTCACTCG

## Data Availability

Not applicable.
